# Non-aneurysmal Subarachnoid Hemorrhage Due to an Aplastic or Twig-Like Middle Cerebral Artery Diagnosed by Cone Beam CT and M1 Selective Angiography: A Case Report

**DOI:** 10.7759/cureus.69285

**Published:** 2024-09-12

**Authors:** Seigo Kimura, Ryokichi Yagi, Keiichi Yamada, Hirokatsu Taniguchi, Masahiko Wanibuchi

**Affiliations:** 1 Neurosurgery, Kouzenkai Yagi Neurosurgical Hospital, Osaka, JPN; 2 Department of Neurosurgery, Osaka Medical and Pharmaceutical University, Takatsuki, JPN

**Keywords:** cone-beam computed tomography, conservative therapy, middle cerebral artery, non-aneurysmal subarachnoid hemorrhage, twig-like mca

## Abstract

A non-aneurysmal subarachnoid hemorrhage (SAH) due to an aplastic or twig-like middle cerebral artery (Ap/T-MCA) is extremely rare. We describe a case of a non-aneurysmal SAH due to an Ap/T-MCA. A 38-year-old female patient experienced a sudden severe headache and was admitted to our hospital. A head CT scan revealed an SAH. Cerebral angiography revealed a non-aneurysmal subarachnoid hemorrhage due to an Ap/T-MCA. The patient was treated conservatively and had a good recovery. Because of the complexity of the vascular structure, we had difficulty in making a diagnosis of our case. Cone beam CT and M1 selective angiography using a microcatheter were useful for the diagnosis of an Ap/T-MCA. An extracranial-intracranial bypass (EC-IC) may effectively treat a non-aneurysmal SAH due to an Ap/T-MCA. However, the evidence of the effectiveness of EC-IC bypass surgeries for non-aneurysmal SAHs due to Ap/T-MCAs is insufficient, and further case accumulation is needed.

## Introduction

An aplastic or twig-like middle cerebral artery (Ap/T-MCA) is a congenital developmental abnormality of the middle cerebral artery (MCA) during embryogenesis [1]. The MCA usually develops through the fusion and regression of twig-like networks in the early embryonic stage [2]. An Ap/T-MCA may result from hypoplasia or the persistence of twig-like networks due to M1 fusion failure [1]. An Ap/T-MCA is a rare lesion found in 0.11%-1.17% of patients undergoing cerebral angiography [1-3]. A non-aneurysmal subarachnoid hemorrhage (SAH) due to an Ap/T-MCA is extremely rare [1,4-6]. Here, we describe a case of a non-aneurysmal SAH due to an Ap/T-MCA. We also report the diagnostic utility of cone beam CT and M1 selective angiography using a microcatheter in its diagnosis.

## Case presentation

**Figure 1:**
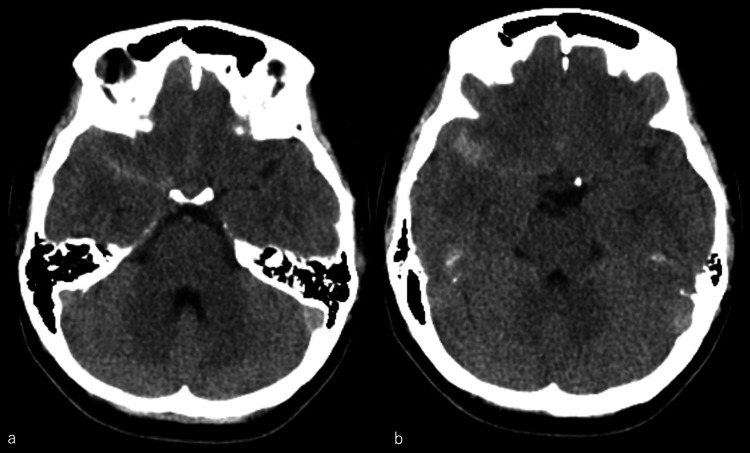
a, b) Head CT revealing a subarachnoid hemorrhage almost localized to the right Sylvian fissure where the plexiform arterial network was observed.

A 38-year-old female patient experienced a sudden severe headache and nausea and was admitted to our hospital for emergency medical assistance. Her medical and family history was insignificant. On arrival, the patient’s Glasgow Coma Scale score was 15 (E4, V5, M6) with no apparent paresis. The blood pressure and the pulse rate were 126/81 mmHg and 93 beats/min, respectively. A head CT scan revealed an SAH (Fisher group 2) almost localized to the right Sylvian fissure with a World Federation of Neurosurgical Societies grade of I and Hunt and Kosnik grade of I (Figures 1a, 1b). 

**Figure 2:**
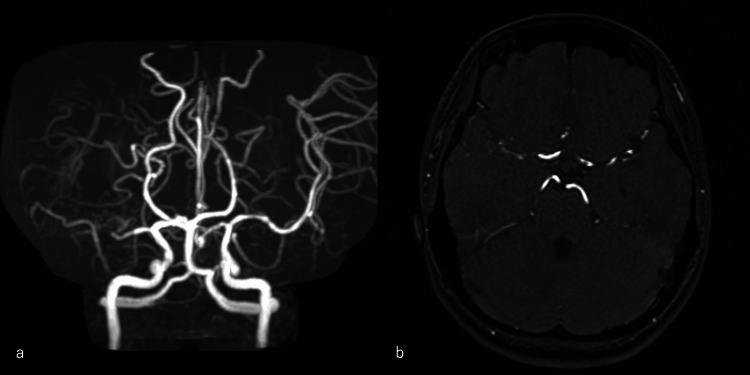
a) Head magnetic resonance angiography (MRA) revealing an unobvious right middle cerebral artery. b) In the original head MRA images, the plexiform arterial network was observed in the subarachnoid space.

Head magnetic resonance angiography (MRA) revealed an unobvious right MCA (Figure 2a). In the original head MRA images, the plexiform arterial network was observed in the subarachnoid space (Figure 2b).

**Figure 3:**
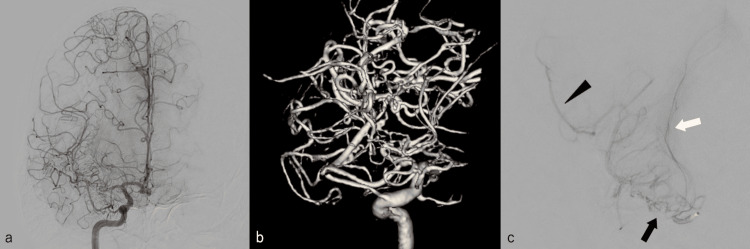
a) Cerebral angiography of the right internal carotid on day one. b) Volume-rendering image of cerebral angiography on day one. c) M1 selective angiography using a microcatheter performed three months after hospital discharge. Black arrow: plexiform arterial network. Black arrowhead: cortical branch of the middle cerebral artery. White arrow: lenticulostriate arteries arose from the plexiform arterial network.

**Figure 4:**
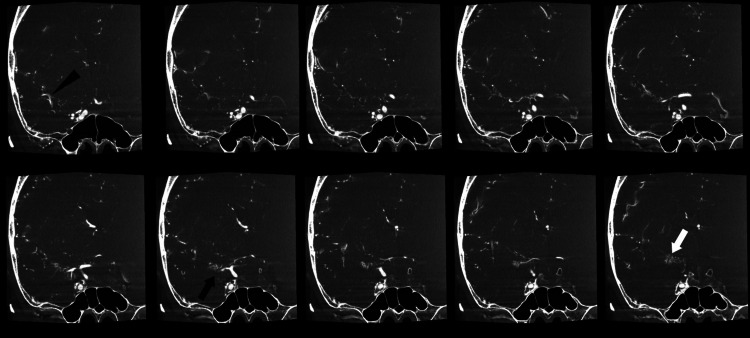
Cone beam CT performed three months after hospital discharge. Black arrow: plexiform arterial network. Black arrowhead: cortical branch of the middle cerebral artery. White arrow: lenticulostriate arteries arose from the plexiform arterial network.

The patient was treated conservatively with blood pressure below 120 mmHg. Cerebral angiography was performed on day one. Right internal carotid angiography (Figure 3a) and volume-rendering imaging (Figure 3b) revealed a plexiform arterial network and cortical branch with a normal vessel diameter. No transdural anastomosis via the external carotid artery was observed. No obvious vascular lesions in the other main vessels were noted. The patient’s symptoms improved, and she was discharged on day 17 with a modified Rankin Scale score of 0. Head magnetic resonance angiography and cerebral angiography performed three months after discharge revealed no significant changes. M1 selective angiography using a microcatheter (Figure 3c) and cone beam CT (Figure 4) revealed that the plexiform arterial network converged peripherally with the cortical branches with a normal vessel diameter. Lenticulostriate arteries arose from the plexiform arterial network. The SAH did not recur within a six-month period after hospital discharge.

## Discussion

In an analysis of 82 cases of Ap/T-MCAs in patients aged 7-81 years (mean 46.9 years), the highest incidence was found in the 40-50 years age group, mostly in East Asia and especially in Japan and Korea [7]. 

The features of Ap/T-MCAs on angiographical imaging are as follows: 1) stenosis or occlusion localized to the unilateral M1 segment, with cortical branches distal to the M1 segment typically appearing normal; 2) other cerebrovascular systems being mostly unaffected; 3) an absence of transdural anastomosis through the external carotid artery but with leptomeningeal collateral circulation; 4) a plexiform arterial network instead of the M1 segment; and 5) lenticulostriate arteries arising from the plexiform arterial network [1,3,8]. Unlike moyamoya disease, an Ap/T-MCA does not feature the progression of lesions, stenosis at the terminal portion of the internal carotid artery, vessel lesions in the posterior cerebral artery, or transdural anastomosis from the external carotid artery. An Ap/T-MCA is predominantly unilateral, and the plexiform arterial network is located in the subarachnoid rather than the intraparenchymal space [1,8]. Our case did not feature stenosis at the terminal portion of the internal carotid artery, vessel lesions in the posterior cerebral artery, or transdural anastomosis from the external carotid artery. The plexiform arterial network of our case was predominantly unilateral and was located in the subarachnoid rather than the intraparenchymal space. The lenticulostriate arteries in our case arose from the plexiform arterial network. Therefore our case exhibited features of an Ap/T-MCA, not those of moyamoya disease.

The possibility of poor-quality angiography because of dissection was also considered. Initial cerebral angiography was not performed with a microcatheter because guiding the microcatheter into the MCA may negatively affect the dissection. Thus, only conventional imaging was performed, and the patient was treated conservatively. Vascular dissection is usually associated with remodeling over time. However, our case did not show remodeling, indicating the possibility of an Ap/T-MCA. The right MCA also exhibited a plexiform arterial network on conventional digital subtraction angiography and three-dimensional imaging. However, because of the complexity of the vascular structure, the convergence of the peripheral cortical branches and the size of their diameter were unclear, making diagnosis difficult.

Follow-up cerebral angiography was performed, with additional cone beam CT and M1 selective angiography using a microcatheter. Cone beam CT is a cross-sectional image. In addition, M1 selective angiography reveals the continuity from the plexiform arterial network to the cortical branch. Cone beam CT and M1 selective angiography revealed a plexiform arterial network in the right M1. The cortical branch where the plexiform arterial network converged was of normal diameter, and lenticulostriate arteries arose from the plexiform arterial network. Therefore, this case was diagnosed as an Ap/T-MCA. Cone beam CT and M1 selective angiography using a microcatheter were useful for the diagnosis of the Ap/T-MCA. According to Fukuda et al. [7], 65% of patients diagnosed with Ap/T-MCAs experienced hemorrhagic strokes, of which 20% experienced ischemic strokes and 15% were asymptomatic. Cerebral aneurysms occur in 40% of Ap/T-MCAs, of which 76% would rupture, the risk of which is high [4]. The thin walls of the plexiform arterial network and the lack of a well-developed muscular layer may increase susceptibility to cerebral aneurysm formation and rupture due to hemodynamic stress [1,3,9]. No cerebral aneurysms were observed in our case; however, the SAH may have been caused by hemodynamic stress to the fragile plexiform arterial network in the subarachnoid space.

In moyamoya disease, long-term hemodynamic stress on the moyamoya vessels may cause bleeding, and the effectiveness of extracranial to intracranial (EC-IC) bypass surgeries in preventing hemorrhagic events has been reported [1,10,11]. Ap/T-MCAs and moyamoya disease have similar pathologies and both may be affected by hemodynamic stress. Takarada et al. [12] performed EC-IC bypass surgeries in patients with Ap/T-MCAs and subarachnoid hemorrhage due to ruptured cerebral aneurysms. Several EC-IC bypass procedures for similar cases have been reported, including cases in which the plexiform arterial network had regressed [13,14,15] and five cases of non-aneurysmal SAHs due to Ap/T-MCAs (Table 1).

**Table 1:** Cases of non-aneurysmal subarachnoid hemorrhage due to aplastic or twig-like middle cerebral artery SAH: subarachnoid hemorrhage; GR: good recovery; N/A: not applicable

Author and year	Case number	Age/Sex	clinical presentation	Side	SAH distribution	Treatment	Outcome
Seo et al. 2012 [1]	Case 1	73/M	SAH	Left	N/A	Conservative	N/A
Tashiro et al. 2016 [4]	Case 2	76/F	SAH	Left	Left Sylvian fissure	Conservative	GR
Tashiro et al. 2016 [4]	Case 3	81/F	SAH	Left	Left temporal lobe	Conservative	GR
Shirokane et al. 2019 [5]	Case 4	64/F	SAH	Bilateral	Left Sylvian fissure	Left direct bypass	GR
Kawakami et al. 2022 [6]	Case 5	67/M	SAH	Left	Left Sylvian fissure	Conservative	GR
Kimura et al.	Present Case	38/F	SAH	Right	Right sylvian fissure	Conservative	GR

In the latter five cases, the distribution of the SAH was localized to the Sylvian fissure or subarachnoid temporal lobe, except in one case in which this information was unknown. Four of the five patients were treated conservatively, and one patient underwent an EC-IC bypass. The disease course of one patient whose SAH distribution was unknown and who was treated conservatively could not be obtained. However, the remaining four patients showed good outcomes, with one patient who underwent an EC-IC bypass and three patients who were treated conservatively, with no recurrence of SAHs. An EC-IC bypass may reduce hemodynamic stress and prevent cerebral aneurysm development and rupture. However, its efficacy in the treatment of a non-aneurysmal SAH due to an Ap/T-MCA is currently unknown. The risk of SAH recurrence and cerebral aneurysm development in Ap/T-MCAs remains. Therefore, continued careful imaging follow-up not only by MRA but also by cerebral angiography as often as possible is considered necessary.

## Conclusions

We reported a case of a non-aneurysmal SAH due to an Ap/T-MCA. Cone beam CT and M1 selective angiography using a microcatheter were useful in the diagnosis of the non-aneurysmal SAH due to an Ap/T-MCA. EC-IC bypass procedures may effectively treat non-aneurysmal SAHs due to Ap/T-MCAs. However, the evidence of the effectiveness of EC-IC bypass procedures for non-aneurysmal SAHs due to Ap/T-MCAs is insufficient, and further case accumulation is needed.
